# No Gentlemen Need Apply

**Published:** 1919-05-31

**Authors:** 


### THE UNPARDONABLE POSTCARD.

### No Gentlemen Need Apply.

WE have sometimes been criticised for remarking that the *personnel* of hospital administration is still weak in the higher qualifications, and have argued from this fact, for it is a fact, that only higher salaries can attract the right type of man for the work. Business experience is necessary, but a new clerk is not an administrator. Energy, commercial knowledge, are necessary; but qualities are needed which no experience in an office by itself can teach. Let us illustrate our case from a twofold instance.

It is the duty of many secretaries to inform medical candidates for house appointments of the result of their application. The right type of man replies by letter, the wrong type of man by a postcard. If the latter should be told that he had thereby been guilty, not merely of inconceivable want of tact, but of an act of rudeness, he would probably reply that he was busy and anxious to save a halfpenny stamp. Now the man who thinks in halfpenny stamps is not a fit and proper man for the post of hospital secretary. Since this habit of replying to medical candidates by postcard is still current, it becomes necessary to show those guilty of it, with all plainness, the unpardonable nature of their offence.

The use of a postcard itself is ungracious, as is apparent from the fact that no gentleman would answer an invitation or acknowledge his pleasure in a recent visit to his hostess by its means. Among people of breeding, even when they are friends, it is used only to express the most formal and trivial details, such as a safe arrival at the end of a journey when there is no time for a proper note. The only excuses for its use are great haste, triviality of news, or close intimacy, such as a line to an old friend making or suggesting a sudden and unexpected appointment. Even in these cases a telegram is more courteous, partly because a telegram considers the convenience of the addressee, and partly because a telegram is at all events private and personal, whereas a postcard invites the attention of any unscrupulous person, indeed of any one, including a servant, through whose hands it chances to pass. There is, in short, no less considerate way of communicating a written message than by a postcard. Consequently only tradesmen at the one extreme and near relatives at the other ever use it.

Now a hospital secretary is not in the position of a near relation to presume to send a postcard to a medical candidate, nor, unless his actions place him there, in that of a tradesman. On the contrary, he replies to such applications in his capacity as the representative of a corporate body, of an institution, whose reputation must be his chief care. Further, the matter which the secretary has to communicate is personal and private to the last degree. It is the exception that a candidate for any post cares to have the fact of his candidature known. To advertise his success by sending him a postcard is treatment so casual, so unconsidered, as to be perilously near a snub. To advertise his failure on a postcard is simply to insult him gratuitously.

If there is any so lacking in delicacy as to think this statement exaggerated, let him put himself in the position of an unsuccessful candidate, for, say, the post of house surgeon. Such a man who hears of his failure by postcard will immediately ask: "What right has this wretched secretary to make my private affairs public property in this fashion? Who is he to go out of his way to annoy a stranger in this way? What right has he to use his position to insult gentlemen whose applications he has publicly invited?" The first result of the postcard has been to make the hospital an enemy for life. For such rudeness is never forgiven. The next thought that will occur to the unsuccessful candidate will be one of relief at having escaped more intimate knowledge of a hospital whose officers have no idea how to behave themselves. "If the secretary," the doctor will say, "is without the elementary notions of good breeding, what must be the atmosphere of the place? Since it takes care to show that gentlemen should not apply, let it be content with bounders." Thus does a hospital earn a bad name; for every post the application for which is acknowledged by postcard will damn the hospital in the eyes of successive generations of medical men.

It needs but little foresight to predict the time when the halfpenny stamp secretary will have ruined the reputation of his hospital, when its staff will begin to become known as second-rate, when a prejudice will have arisen against it, when support will be difficult to secure, till at last some trouble, perhaps a scandal, will bring matters to a very disagreeable head.

It is the plain duty of every chairman who discovers that his secretary is replying to candidates by postcards to give him a severe reprimand, and an experienced chairman will see, from the way in which that reprimand is taken, whether the secretary is worth another trial. The use of a postcard reveals such a defect of breeding, such inconceivable want of tact, even of the manners of business itself, that a word of excuse must put the secretary permanently out of court. Only the fullest apology can save his character. For only a full apology can show that the unfortunate man realises what he has done. To realise is to repent, and without realisation there can be no repentance or guarantee of avoiding similar errors.

